# MeshLifter: Weakly Supervised Approach for 3D Human Mesh Reconstruction from a Single 2D Pose Based on Loop Structure

**DOI:** 10.3390/s20154257

**Published:** 2020-07-30

**Authors:** Sunwon Jeong, Ju Yong Chang

**Affiliations:** Department of Electronics and Communication Engineering, Kwangwoon University, Seoul 01897, Korea; bmycrew@kw.ac.kr

**Keywords:** 3D human mesh reconstruction, 3D human pose estimation, deep neural network, weakly supervised learning

## Abstract

In this paper, we address the problem of 3D human mesh reconstruction from a single 2D human pose based on deep learning. We propose MeshLifter, a network that estimates a 3D human mesh from an input 2D human pose. Unlike most existing 3D human mesh reconstruction studies that train models using paired 2D and 3D data, we propose a weakly supervised learning method based on a loop structure to train the MeshLifter. The proposed method alleviates the difficulty of obtaining ground-truth 3D data to ensure that the MeshLifter can be trained successfully from a 2D human pose dataset and an unpaired 3D motion capture dataset. We compare the proposed method with recent state-of-the-art studies through various experiments and show that the proposed method achieves effective 3D human mesh reconstruction performance. Notably, our proposed method achieves a reconstruction error of 59.1 mm without using the 3D ground-truth data of Human3.6M, the standard dataset for 3D human mesh reconstruction.

## 1. Introduction

Nowadays, intelligent sensors such as the Microsoft Kinect can perform human body motion recognition and have been successfully used in various applications such as human–computer interaction, virtual reality, and intelligent robots. Moreover, the recent rapid development of data-driven approaches, including deep learning, has made it possible to use more general red, green, and blue (RGB) image sensors for human body motion analysis than depth sensors such as the Microsoft Kinect. In this paper, we address the problem of 3D human pose and shape reconstruction using a single monocular RGB sensor.

In the area of computer vision, research on 2D and 3D human pose estimation from a single RGB image have been improved considerably in recent years [1,2]. However, these studies only generate sparse keypoints of the human subject. We need dense shape information on the target subject to obtain a deeper understanding of the human image. Most recent studies use the 3D morphable model (3DMM) called skinned multi-person linear model (SMPL) [3] to reconstruct the 3D shape of a person from an image. SMPL parameterizes the variation of the 3D human mesh using low-dimensional latent variables, such as pose and shape.

The recent 3D human body mesh reconstruction method based on SMPL is typically classified into two classes. The first is an optimization-based method that minimizes the energy function to fit the parameterized body model to the 2D features extracted from the input image. This method has the advantage of accurately obtaining a human body mesh without training with a dataset. However, this method has the following disadvantages. First, the optimization algorithm is sensitive to the initial point. If an appropriate initial point is not given, the optimization algorithm may fall into the local minima, which prevents a satisfactory reconstruction result. Second, the optimization process is generally very slow.

Methods using deep-learning-based regression networks have been proposed recently to overcome the disadvantages of the optimization-based approach. Deep learning networks run faster than optimization-based methods but have the following disadvantages. In most deep-learning-based methods, the network is trained using many pairs of inputs and outputs. Therefore, training a 3D human mesh reconstruction network based on SMPL requires a large dataset that includes many input images and their corresponding SMPL parameters. However, obtaining ground-truth SMPL parameters is generally very difficult. Therefore, most existing studies train the network indirectly using 3D poses instead of SMPL parameters. However, even 3D human poses are not easy to acquire in an in-the-wild environment. Hence, in this study, we propose a method to train a 3D human mesh reconstruction model without paired 2D and 3D data to solve the 3D data acquisition difficulty. Our method includes the following two contributions.

The first is MeshLifter, which is a deep learning model that outputs a 3D human mesh from an input 2D human pose and has the encoder–decoder structure. The encoder composed of residual blocks and fully connected layers outputs SMPL parameters from the input 2D human pose. The decoder with SMPL layer outputs 3D human mesh from the SMPL parameters generated from the encoder. A 3D pose can be also obtained from a 3D mesh using the pre-trained regression matrix included in the SMPL layer. Therefore, we can obtain the 3D mesh and 3D pose simultaneously from the input 2D human pose through the MeshLifter.

The second is a weakly supervised learning method based on a loop structure. The loop structure was first proposed in [4] to solve projection ambiguity where multiple 3D poses can be mapped to one 2D human pose. In [4], through the proposed loop structure, a lifting network that estimates a 3D pose from a 2D pose is trained without a 3D pose dataset. Our research goes further and proposes a method to learn MeshLifter, a model that can estimate 3D pose and 3D mesh from a single 2D pose. In the loop structure proposed in this study, 3D mesh lifting from a 2D pose through the MeshLifter and random rotation and 2D projection of a 3D pose computed through the MeshLifter are repeated, thereby providing a novel loop loss using only 2D pose data.

**Figure 1:**
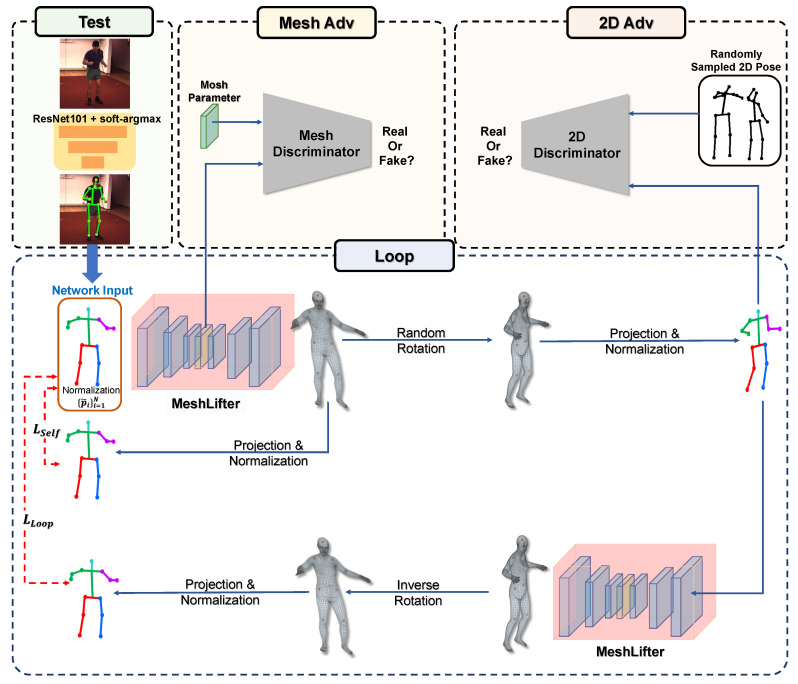
Overview of the proposed method.

We reconstruct the 3D mesh by feeding the 2D pose estimated from the input image and not the ground-truth 2D pose into the MeshLifter to show the effectiveness of the proposed method. We use a general 2D human pose estimator based on a convolutional neural network (CNN). Through this experiment, we show that the MeshLifter can successfully reconstruct a 3D human mesh from a noisy input 2D human pose. In addition, through experiments using various datasets, such as Human3.6M [5], MPI-INF-3DHP [6], and MPII [7], we show that the proposed method achieves comparable performance with existing state-of-the-art methods. An overview of the proposed method is illustrated in Figure 1.

## 2. Related Work

Recent 3D human body mesh reconstruction studies can be grouped into two main categories. The first is optimization-based methods that minimize the energy function to fit the parameterized body model to 2D features extracted from the input image. The second method regresses the parameters of the 3DMM directly from the input 2D feature through the deep neural network.

### 2.1. Optimization-Based SMPL Parameter Fitting

In [8], the 3D human mesh is reconstructed through the following two-step process. First, 2D joints are extracted from the input image through CNN. SMPL parameters are obtained through optimization of the proposed energy function using the extracted 2D joints. [9] is an extension of [8], which estimates SMPL parameters by minimizing the energy function based on body part segments and joint annotations. These optimization-based methods generally have high computational complexity and have the disadvantage of being easy to fall into local minima if a good initial point is not given.

### 2.2. Deep-Learning-Based SMPL Parameter Regression

Recently, many methods for directly regressing SMPL parameters using deep neural networks have been proposed. Kanazawa et al. [10] proposed a regression network that directly estimates SMPL parameters from image features extracted from an input image. This method also regularizes the output SMPL parameters through additional adversarial learning to enforce plausibility of the resultant 3D mesh. In [11], the extracted image feature and template mesh are fed into the graph CNN and the graph CNN generates intermediate mesh vertices. The shape and pose parameters are regressed from the intermediate mesh vertices, which results in the finer human mesh. In [12], a data fusion module that allows not only RGB images but also RGB-D images to be used as input has been proposed. The module enables more robust mesh reconstruction. In addition, a probabilistic learning process has been proposed to simulate missing data in the process of using RGB-D data, which allows various datasets to be used for learning.

In [13], a method in which optimization and deep-learning-based regression are combined was proposed. In this method, the deep network regresses the SMPL parameter, which is used as the initial value of the iterative optimization routine [8] for fitting the SMPL model to 2D joints within the training loop. The SMPL parameter computed through optimization is used to explicitly supervise the deep network. The proposed method currently shows state-of-the-art performance and has been proved to be effective especially when 3D ground-truth is lacking or not available.

The proposed method belongs to a deep-learning-based direct regression approach. Therefore, it does not perform the optimization process, which requires proper initialization and has relatively high computational complexity. Also, unlike many methods that belong to the direct regression approach, our method does not require paired 2D and 3D data for training. Finally, our MeshLifter can use either a 2D human pose or an RGB image as input, unlike most other methods that only use RGB images to obtain a 3D human mesh. This flexibility in input makes the proposed method easy to use. A comparison of 3D human body mesh reconstruction methods, including the proposed method, is presented in Table 1.

## 3. Proposed Method

### 3.1. MeshLifter

**Figure 2:**
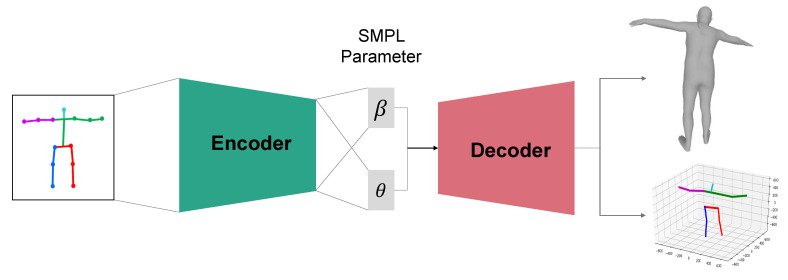
Overview of the MeshLifter.

MeshLifter consists of an encoder and a decoder and outputs a 3D mesh $M\in R^{3\times V}$ of V(=6890) vertices and a 3D pose $P\in R^{3\times N}$ from an input normalized 2D pose vector $\tilde{p}\in R^{2N}$ composed of pixel coordinates of N(=14) joints. Figure 2 shows an overview of the MeshLifter structure composed of the sequential combination of encoder and decoder.

#### 3.1.1. SMPL

We use 3DMM to represent the 3D human shape, in which 3D geometry and texture variations of human subjects are represented by a low-dimensional parameter vector. SMPL is a 3DMM proposed in [3] to represent human body mesh. In SMPL, the principal component analysis is applied to thousands of 3D body scans to determine shape parameters $\beta \in R^{10}$ and pose parameters $\theta \in R^{72}$. First, the shape parameter β represents shape variations that the human body can have, such as fatness. The pose parameter θ represents a 72-dimensional (3×23+3=72) vector that parameterizes the global rotation of the body mesh and the local rotations of 23 joints in an axis-angle fashion. The joints of the body mesh rotate according to the pose parameter, which enables a representation of the body shape that corresponds to various postures by deforming the mesh with the shape parameters.

#### 3.1.2. Encoder

The input 2D pose ${\left\{p_{i}\right\}}_{i=1}^{N}$ $\left(p_{i}\in R^{2}\right)$ is first converted to the normalized 2D pose ${\left\{{\tilde{p}}_{i}\right\}}_{i=1}^{N}$ with zero mean and unit variance. The normalized 2D pose vector $\tilde{p}=\left[{\tilde{p}}_{1};\ldots ;{\tilde{p}}_{N}\right]$ is then used as the input of the encoder:

(1) $${\tilde{p}}_{i}=\frac{p_{i}-m}{\sigma },$$

where m and σ denote mean vector and standard deviation, respectively, and are calculated as follows:

(2) $$m=\sum_{i=1}^{N}\frac{p_{i}}{N},$$

(3) $$\sigma =\sqrt{\sum_{i=1}^{N}\frac{∥p_{i}{-m∥}^{2}}{N}}.$$

From the input normalized 2D pose vector, the encoder outputs low-dimensional feature vectors Θ=(θ,β) that control the human mesh. θ and β represent the SMPL pose and shape parameters, respectively, and are fed into the decoder.

#### 3.1.3. Decoder

The decoder generates the 3D mesh *M* from the shape and pose parameters output from the encoder based on the SMPL model. The decoder also computes the 3D pose P from the reconstructed mesh by using the pretrained regression matrix $W\in R^{V\times N}$ as follows:

(4) P=MW.

The decoder consists of only differentiable operations and thus, the MeshLifter that contains it can be trained based on back-propagation.

### 3.2. Weakly Supervised Learning Based on Loop Structure

The ground-truth dataset is required for the input 2D pose and its corresponding output 3D pose or SMPL parameter to train the MeshLifter that outputs 3D human mesh and pose in a supervised learning manner. However, acquiring ground-truth data for 3D pose and SMPL parameters is generally not easy. Therefore, we propose the following method to learn the MeshLifter from a large 2D pose dataset in a weakly supervised manner using the loop structure introduced for unsupervised 3D human pose estimation in [4].

**Figure 3:**
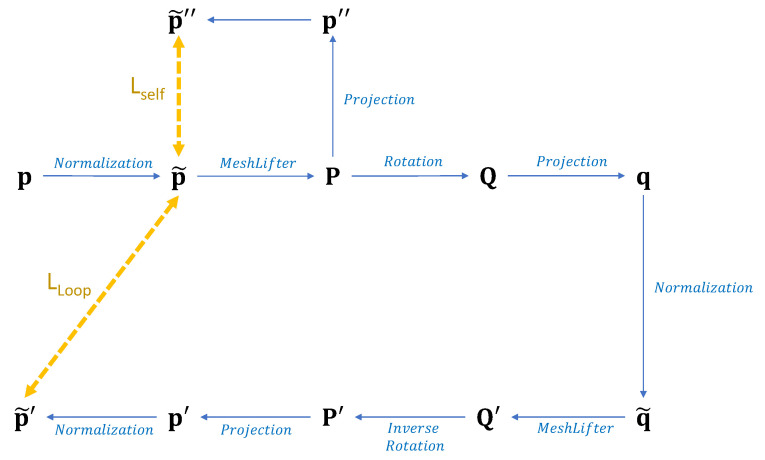
Overview of the loop structure.

**Figure 4:**
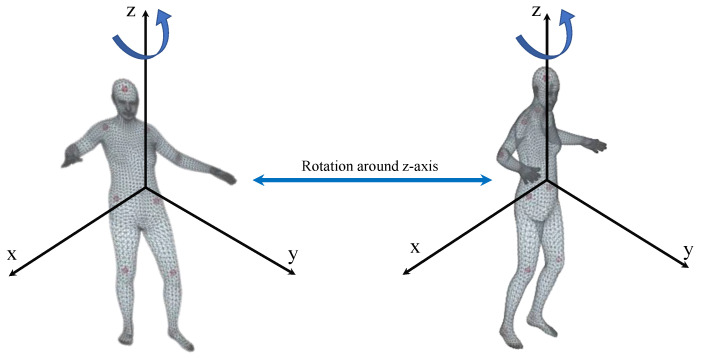
Rotation around *z*-axis.

First, the overview of the loop structure is shown in Figure 3. The loop structure consists of two MeshLifters that share parameters, random rotation, and its inverse rotation, projection, and normalization processes. Random rotation rotates the 3D pose around the z-axis through the angle sampled from $\left[-60^{\circ },60^{\circ }\right]$, as shown in Figure 4. Projection converts the 3D pose into the 2D pose according to the orthographic projection model.

After the input 2D human pose p is normalized, it is fed into the MeshLifter to output the 3D pose P. A rotated 3D pose Q is obtained by applying a random rotation *R* to the output 3D pose P. Q is projected onto the 2D image plane through orthographic projection. After the projected 2D pose q is normalized by Equation (1), it is fed into the MeshLifter to output $Q^{\prime }$. $P^{\prime }$ is obtained by applying the inverse transform $R^{-1}$ of the rotation *R* to the 3D pose $Q^{\prime }$. Finally, $P^{\prime }$ is projected onto the 2D image plane and then normalized to obtain $p^{\prime }$. If the MeshLifter is learned correctly, the normalized 2D pose p and the 2D pose $p^{\prime }$ obtained through the loop structure should be the same. Therefore, we define the loss $L_{Loop}$ based on L1 norm as follows:

(5) $$L_{Loop}={∥\tilde{p}-{\tilde{p}}^{\prime }∥}_{1}.$$

For the successful learning of the MeshLifter, the self-supervision loss $L_{Self}$ that is motivated by self-supervised learning is introduced in addition to $L_{Loop}$. $L_{Self}$ is defined as the difference between the input normalized 2D pose $\tilde{p}$ and the 2D pose ${\tilde{p}}^{\prime \prime }$ obtained by projecting the 3D pose P from the first MeshLifter onto 2D image plane and normalizing the result:

(6) $$L_{Self}={∥\tilde{p}-{\tilde{p}}^{\prime \prime }∥}_{1}.$$

This provides the additional constraint so that the MeshLifter produces a more accurate 3D pose.

### 3.3. Adversarial Training

#### 3.3.1. Mesh Adversarial Training

The MeshLifter can be trained using the losses proposed above. However, these losses do not supervise the output SMPL parameters directly and thus, these losses alone cannot prevent the MeshLifter from outputting anthropometrically implausible body meshes. Therefore, we prevent this through adversarial training introduced in [10]. We consider the encoder of the MeshLifter as generator G and perform adversarial training using the discriminator network D for SMPL parameters. Discriminator D distinguishes whether the SMPL parameter output from the encoder corresponds to a real or fake human mesh, and generator G is trained to output a 3D shape and pose parameter that represents a plausible human mesh to deceive the discriminator.

We use different networks as discriminators for pose θ and shape β parameters, both of which are denoted by $D_{\theta }$ and $D_{\beta }$, respectively. $D_{\beta }$ outputs the probability that input β corresponds to the real human mesh. $D_{\theta }$ consists of a total of K+1 discriminators that include *K* discriminators to learn the possible rotation range of joints, and a discriminator that determines the holistic plausibility of the mesh from full pose parameters. Here, K(=23) is the number of joints in the SMPL model, and is different from *N* which is the number of joints for the 2D and 3D pose. The loss function for generator G is as follows:

(7) $$L_{Adv_{mesh}}\left(G\right)=\sum_{i}E_{\Theta ∼p_{G}}\left[{\left(D_{i}\left(G\left(\tilde{p}\right)\right)-1\right)}^{2}\right],$$

where Θ=(θ,β) and *i* represent the output SMPL parameters of the encoder and the index of the discriminator, respectively. The Mosh dataset [14] with ground-truth SMPL parameters is used to train the discriminator. The discriminator is trained to determine the parameter output from the encoder as fake and determine the parameter of the Mosh dataset as real. The cost function for discriminator learning is as follows:

(8) $$L_{Disc_{mesh}}\left(D_{i}\right)=E_{\Theta ∼p_{mosh}}\left[{\left(D_{i}\left(\Theta \right)-1\right)}^{2}\right]+E_{\Theta ∼p_{G}}\left[{\left(D_{i}(G\left(\tilde{p}\right)\right)}^{2}\right].$$

#### 3.3.2. 2D Pose Adversarial Training

Additional adversarial training is performed using 2D human pose data, of which the ground-truth data is relatively easy to obtain. To this end, we consider the 2D pose $\tilde{q}$ obtained by applying random rotation and projection to the 3D pose generated from the first MeshLifter as the output of the generator. The discriminator is trained to judge the $\tilde{q}$ produced by the generator as fake, and to judge the 2D pose $\tilde{r}$ obtained through random sampling and normalization from the ground-truth 2D human pose dataset as real. Therefore, the loss function for the generator is as follows:

(9) $$L_{Adv_{2d}}\left(G\right)=\sum_{i}E_{\tilde{q}}\left[{\left(D_{i}\left(\tilde{q}\right)-1\right)}^{2}\right],$$

and the loss function for the discriminator is as follows:

(10) $$L_{Disc_{2d}}\left(D\right)=E_{\tilde{r}}\left[{\left(D(\tilde{r})-1\right)}^{2}\right]+E_{\tilde{q}}\left[D{\left(\tilde{q}\right)}^{2}\right].$$

### 3.4. Regularization

In addition to adversarial training, we adopt a regularization term for the plausibility of the reconstructed human mesh. The regularization term for this is defined as follows:

(11) $$L_{Reg}={∥\beta -\beta_{0}∥}_{1},$$

where β is the shape parameter generated from the first MeshLifter and $\beta_{0}$ is the shape parameter of the template mesh.

Therefore, the final loss function for training the proposed MeshLifter in a weakly supervised manner is as follows:

(12) $$L_{Total}=L_{Loop}+\omega_{1}L_{Self}+\omega_{2}L_{Adv_{mesh}}+\omega_{3}L_{Adv_{2d}}+\omega_{4}L_{Reg},$$

where $\omega_{1}$, $\omega_{2}$, $\omega_{3}$, $\omega_{4}$ are weights that control the relative importance of each loss constituting the total loss function. We set $\omega_{1}$, $\omega_{2}$, $\omega_{3}$, $\omega_{4}$ to 1.0, 2.0, 0.1, 0.05 in all our experiments, respectively. These numbers for weights were determined by the following simple greedy search. We first initialize the four weights. Then, after fixing the three weights, the optimal value for the other weight is selected using a small number of pre-sampled candidates. This process is repeated for each of the four weights.

## 4. Experiments

### 4.1. Datasets

The Human3.6M [5] and MPI-INF-3DHP [6] datasets containing RGB human images and corresponding ground-truth 3D human poses are used for training and evaluation of the proposed method. The Mosh [14] dataset containing only the ground-truth SMPL parameters without RGB images is also used for discriminator learning. The Human3.6M dataset [5] provides 3.6 million 2D and 3D human poses and their corresponding RGB images. To construct the dataset, 17 actions (e.g., discussion, smoking, taking the photo, …) of 11 subjects were acquired through a motion capture system using four cameras. We used the subjects S1, S5, S6, S7, and S8 for training and the subjects S9 and S11 for testing according to the conventional protocol. We also sampled one frame every five frames and used it for an experiment to reduce the redundancy of the dataset. The MPI-INF-3DHP dataset [6] consists of approximately 100,000 learning images acquired through a markerless motion capture system indoors and approximately 3000 test images acquired indoors and outdoors. All images in the MPI-INF-3DHP dataset are annotated with 3D human poses. The Mosh dataset [14] was constructed by converting 3D human poses of subjects captured using a marker-based motion capture system into SMPL parameters. Approximately 410,000 SMPL parameters were used for our experiment. Lastly, the MPII dataset [7] containing in-the-wild images is used for the qualitative evaluation of the proposed method. The MPII dataset cannot be used for quantitative evaluation because only 2D poses are annotated. The descriptions of the datasets used in our experiment are summarized in Table 2.

### 4.2. Evaluation Metrics

Under the perspective projection assumption, the 3D shape can be reconstructed only up to a scale factor. Therefore, the proposed method cannot be used to determine the actual body size of the human subject. In consideration of this, we use the reconstruction error, which computes the mean per joint position error (MPJPE) after adjusting the scale and global rotation of the predicted 3D pose and ground-truth 3D pose, according to the Procrustes analysis [15], as the evaluation metric. MPJPE is defined as the average Euclidean distance between the predicted joint $P_{i}$ and grount-truth joint $P_{i}^{*}$ as follows:

(13) $$MPJPE=\frac{1}{N}\sum_{i=1}^{N}{∥P_{i}-P_{i}^{*}\;∥}_{2},$$

where *i* denotes the index of the joint.

### 4.3. Implementation Details

**Figure 5:**
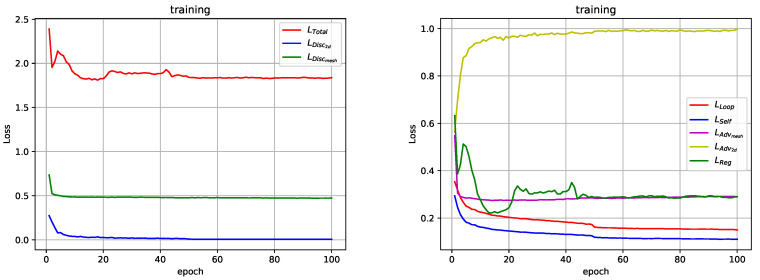
Curves of all our losses during training.

Our code is released at https://github.com/sunwonlikeyou/MeshLifter. The python 3.6 and PyTorch 1.2.0 [16] are used to implement the proposed method. The initial learning rate and the number of epochs are set to $1\times 10^{-4}$ and 100, respectively, to learn the MeshLifter and discriminator. The learning rate decays with a rate of 0.1 after the 50th epoch. Figure 5 shows the curves of all our losses during training. We can observe that the losses except $L_{Adv_{2d}}$ are minimized and converged. One exception, $L_{Adv_{2d}}$, increases with epoch and converges to a value of 1.0. It indicates that the generator, the MeshLifter, fails to produce realistic results that can deceive the 2D pose discriminator. Nevertheless, according to the ablation study in Section 4.4, $L_{Adv_{2d}}$ significantly improves the quantitative performance of the proposed method. We believe that this is because $L_{Adv_{2d}}$ works effectively as a kind of regularization term for plausible 2D poses. The implementation details for MeshLifter and discriminators are as follows.

MeshLifter has an encoder–decoder structure. The encoder consists of linear layer, ReLU [17], dropout [18], batch-norm [19], and residual connection [20]. The decoder includes an SMPL layer composed of only differentiable operations and thus, its parameters can be updated through back-propagation.

Discriminator for SMPL parameter is composed of two networks, $D_{\theta }$ and $D_{\beta }$, that correspond to pose and shape parameters as described in Section 3. $D_{\beta }$ includes two fully connected layers composed of 10 and 5 hidden units and one ReLU layer. $D_{\theta }$ includes a Rodrigues layer, two convolutional layers, and two branches. The Rodrigues layer converts a pose parameter expressed in the axis-angle format to a 3×3 rotation matrix according to the Rodrigues formula. The two convolutional layers consist only of 1×1 size convolution filters, and the number of input and output channels is (9,32) and (32,32), respectively. The first branch contains fully connected layers composed of 736, 1024, and 1024 hidden units. The second branch contains fully connected layers with 32 hidden units for all joints.

Discriminator for 2D pose consists of two consecutive residual blocks and two fully connected layers for input and output of the network. Each residual block includes fully connected layer with 3000 hidden units, batch-norm, dropout, ReLU, and residual connection.

The 2D pose predicted from the RGB image using CNN is used as the input of MeshLifter for a fair comparison with previous methods. The ResNet101 [20] model is used as a backbone network for 2D pose estimation, and the last layer is modified to output a heatmap of 64×64 resolution. We also added the soft-argmax layer [21] to obtain continuous 2D joint coordinates free from quantization error from the heatmap. The network outputs the 2D human pose p from an input RGB image of 256×256 size. Table 3 shows the performance of the 2D human pose estimation.

### 4.4. Ablation Study

An ablation study is performed to investigate the effects of the proposed losses on the performance of our model, and Table 4 shows its quantitative results. The MeshLifter trained with only $L_{Self}$ is considered as the baseline. Loop, Mesh, 2D, and Reg indicate $L_{Loop}$, $L_{Adv_{mesh}}$, $L_{Adv_{2d}}$, and $L_{Reg}$ are used to learn the MeshLifter, respectively.

**Figure 6:**
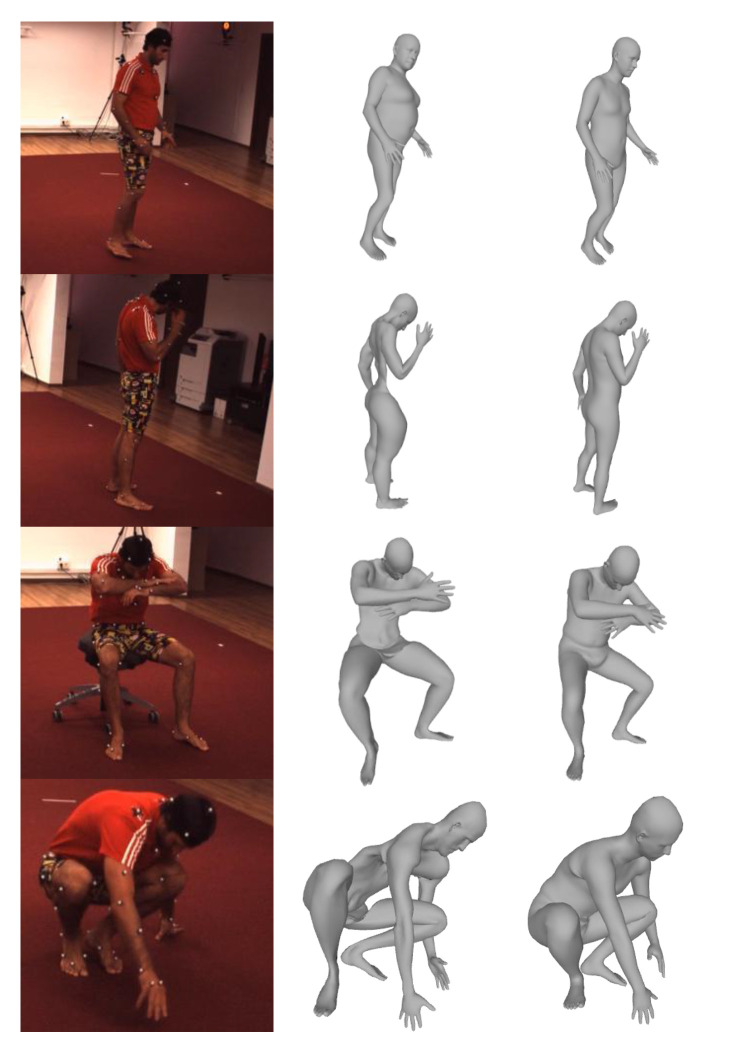
Input images (*left*), and the reconstruction results of using (*right*) and not using (*middle*) the regularization term.

Table 4 shows that $L_{Loop}$, $L_{Adv_{mesh}}$, and $L_{adv_{2d}}$ are significantly helpful for improving the performance of the MeshLifter. According to Table 4, the results obtained using all loss functions except $L_{Reg}$ show the best quantitative performance. However, Figure 6 shows that $L_{Reg}$ plays an important role in the qualitative performance of the reconstructed human mesh. The left, middle, and right columns in Figure 6 show the input image, the 3D mesh output by the MeshLifter trained without $L_{Reg}$, and the 3D mesh output by the MeshLifter trained with $L_{Reg}$, respectively. The use of $L_{Reg}$ prevents the monstrous mesh output and helps in reconstructing the anthropometrically plausible human mesh.

### 4.5. Quantitative Result

Table 5 and Table 6 provide the quantitative results of the proposed method and recent existing methods for the Human3.6M and MPI-INF-3DHP datasets, respectively. They show the proposed method achieved the state-of-the-art performance among methods that do not use 3D pose data as direct supervision for learning. This result shows that the proposed weakly supervised method based on the loop structure effectively learns our MeshLifter.

### 4.6. Qualitative Result

**Figure 7:**
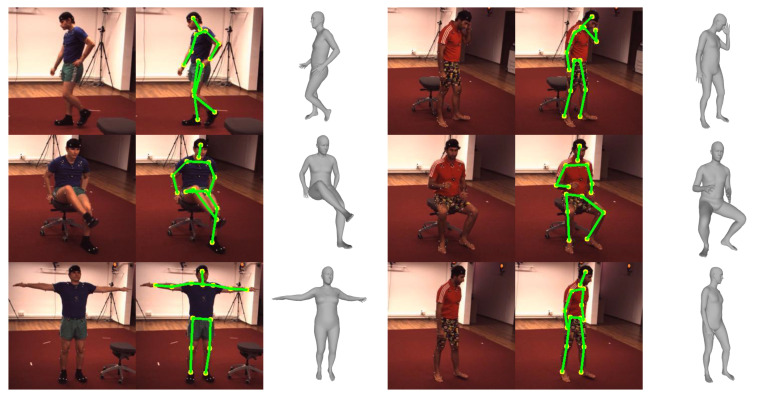
Qualitative results on the Human3.6M dataset.

**Figure 8:**
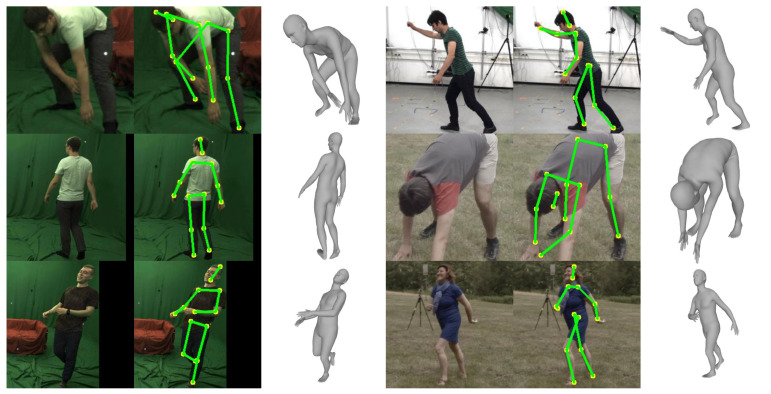
Qualitative results on the MPI-INF-3DHP dataset.

**Figure 9:**
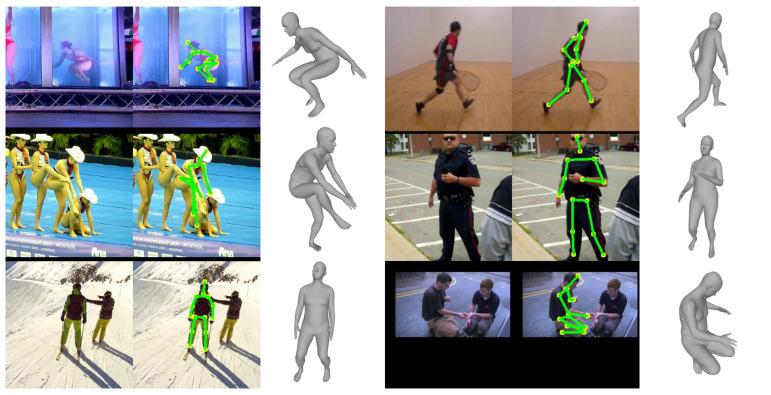
Qualitative results on the MPII dataset.

Figure 7, Figure 8 and Figure 9 show the qualitative results of the proposed method for Human3.6M, MPI-INF-3DHP, and MPII datasets, respectively. The figures show that the proposed method can successfully reconstruct 3D human meshes from various input images acquired in controlled and in-the-wild environments.

### 4.7. 3D Hand Mesh Reconstruction

Additional experiments for hand mesh reconstruction are conducted to investigate the general applicability of the proposed method. To this end, we used MANO [24], a 3DMM for human hands, as a decoder for the MeshLifter. For training and evaluation, we used the Rendered Handpose Dataset (RHD) [25], which includes 24,619 training images and 1459 test images. In the case of hands, a dataset that provides ground-truth 3DMM parameters, such as the Mosh dataset for the body, is not available, and thus, the mesh adversarial training in Section 3.3 cannot be performed. Therefore, we introduce the following regularization term that constrains pose and shape parameters together:

(14) $$L_{Reg_{mano}}={∥\Theta_{h}-\Theta_{0}∥}_{1},$$

where $\Theta_{h}=\left[\theta_{h};\beta_{h}\right]\in R^{58}$ is the MANO shape and pose parameters output from the encoder, and $\Theta_{0}=\left[\theta_{0};\beta_{0}\right]$ is the shape and pose parameters of the template hand mesh. Finally we train the MeshLifter for hands using the following loss function:

(15) $$L_{Total}=L_{Loop}+\omega_{1}L_{Self}+\omega_{2}L_{Adv_{2d}}+\omega_{3}L_{Reg_{mano}},$$

where $\omega_{1}$, $\omega_{2}$, and $\omega_{3}$ are set to 1.0, 0.3, and 0.1, respectively.

**Figure 10:**
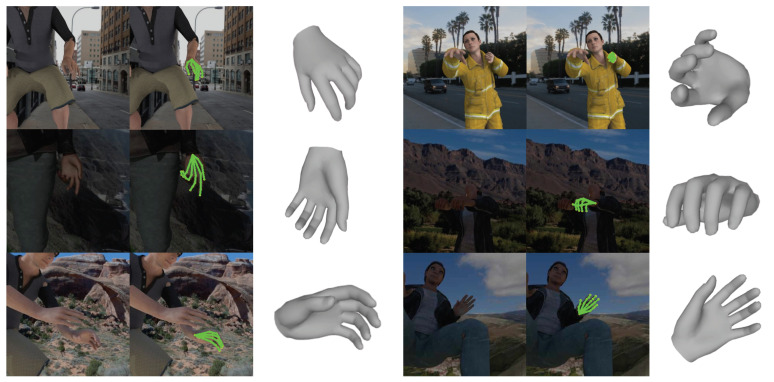
Qualitative results on the RHD dataset.

Table 7 shows a quantitative comparison between the proposed method and existing studies for 3D hand pose estimation. As an evaluation metric for comparison, the reconstruction error is used as in the body. Table 7 shows that the proposed method does not achieve the best performance quantitatively. However, all methods except the proposed method train the network in a supervised manner, and output only sparse 3D hand joints. Meanwhile, the proposed method generates a dense 3D hand mesh in a weakly supervised fashion, which shows the effectiveness of the proposed method. In addition, Figure 10 shows that the proposed method performs qualitatively successful 3D hand mesh reconstruction.

### 4.8. Discussion

In this subsection, we present the usability of the proposed method, limitations, and future works to overcome them. Our proposed method can reconstruct the 3D mesh of the target human object in the form of SMPL parameters. By using the Equation (4), 3D joints can also be obtained from the reconstructed mesh. This 3D skeleton information is used for gesture or action recognition and can be applied to various fields such as human–computer interaction and visual surveillance. Meanwhile, the SMPL parameters reconstructed by the proposed method directly include 3D rotation information of limbs that make up the body beyond merely 3D coordinates of body joints. The rotation information enables motion retargeting between characters and can be used for computer graphics and augmented/virtual reality.

The proposed method uses a single 2D human pose to reconstruct the 3D mesh of the target person. This 2D pose alone does not provide enough information to obtain reliable body shape information, so the proposed method relies heavily on the regularization term to estimate the SMPL shape parameters encoding human body shape information. Therefore, we plan to investigate how additional image features other than 2D poses can have an advantage in estimating shape parameters. Also, the proposed 3D human reconstruction method relies on a single 2D pose or a single RGB image. It makes the temporal prior, which can alleviate the ambiguity of the 3D reconstruction problem, unavailable in the proposed method. It also makes the proposed method produce results that lack temporal consistency when applied to an input 2D pose or image sequence. Therefore, our next future work is to extend the proposed method to 2D pose sequence input and pursue a method in which the proposed model can adopt the temporal prior.

## 5. Conclusions

In this study, we addressed the problem of reconstructing a 3D human mesh from a single 2D human pose. We proposed the MeshLifter, a network that can output 3D mesh and 3D pose from a single 2D pose, and the loop-structure-based method to learn it in a weakly supervised manner. We confirmed that the proposed method efficiently generates a 3D human mesh from a 2D human pose without direct 3D supervision through various experiments and showed that it has comparable performance to the state-of-the-art methods. The MeshLifter trained by our proposed weakly supervised learning method achieves a reconstruction error of 59.1mm for the Human3.6M dataset, the standard dataset for 3D human mesh reconstruction. Through additional experiments, we also showed that our method could be used to reconstruct human hands.

## Figures and Tables

**Table 1:** Comparison of our proposed method with other previous methods for 3D human body mesh reconstruction. “Optimization” indicates that the method depends on the optimization process, which requires parameter initialization and is generally slow. “Regression” indicates that the method is a deep neural network that requires large-scale training data. “Paired 2D–3D” indicates that paired 2D and 3D data should be used for training. “2D pose input” indicates that a 2D human pose can be used as the input to the method instead of an red, green, and blue (RGB) image.

Method	Optimization	Regression	Paired 2D–3D	2D Pose Input
SMPLify [8]	√			√
UP-3D [9]	√			
HMR [10]		√		
CMR [11]		√	√	
RGB-D [12]		√	√	
SPIN [13]	√	√	
Ours		√		√

**Table 2:** Description of the datasets used in our experiment.

Dataset	Human3.6M [5]	MPI-INF-3DHP [6]	Mosh [14]	MPII [7]
Data acquisition	Marker-based motion capture	Marker-less motion capture	Marker-based motion capture	YouTube search
2D image	√	√		√
2D human pose	√	√		√
3D human pose	√	√	√	
SMPL parameters			√	
Number of subjects	11	8	39	40K
Number of examples	3.6M	100K	410K	40K
Purpose of use	Training and evaluation	Training and evaluation	Adversarial training	Qualitative evaluation

**Table 3:** Performance of 2D human pose estimation. The numbers denote mean Euclidean distances in pixels.

Dataset	Pixel Error
Human3.6M	3.2
MPII	6.3

**Table 4:** Ablation experiments with various combinations of losses. The numbers denote reconstruction errors in mm.

Loss Variations	Reconstruction Error
Self (baseline)	157.0
Self + Loop	136.1
Self + Loop + Mesh	83.5
Self + Loop + Mesh + 2D	58.8
Self + Loop + Mesh + 2D + Reg	59.1

**Table 5:** Quantitative results of the proposed model and the existing state-of-the-art methods for the Human3.6M dataset. The numbers denote reconstruction errors in mm.

Method	Reconstruction Error
SMPLify [8]	82.0
Pavlakos et al. [22]	75.9
HMR-unpaired [10]	66.5
SPIN-unpaired [13]	62.0
Ours	**59.1**

**Table 6:** Quantitative results of the proposed model and the existing state-of-the-art methods for the MPI-INF-3DHP dataset. The numbers denote reconstruction errors in mm.

Method	Reconstruction Error
HMR-unpaired [10]	113.2
VNect [23]	98.0
Ours	96.0
SPIN-unpaired [13]	**80.4**

**Table 7:** Quantitative comparison with existing methods on the Rendered Handpose Dataset (RHD) dataset. The numbers denote reconstruction errors in mm.

Method	Reconstruction Error
Zimmermann and Brox [25]	30.42
Yang and Yao [26]	19.95
Spurr et al. [27]	19.73
Yang et al. [28]	**13.14**
Ours	14.02
